# Impaired Inhibitory Control in Recreational Cocaine Users

**DOI:** 10.1371/journal.pone.0001143

**Published:** 2007-11-07

**Authors:** Lorenza S. Colzato, Wery P. M. van den Wildenberg, Bernhard Hommel

**Affiliations:** 1 Institute for Psychological Research and Leiden Institute for Brain and Cognition, Leiden University, Leiden, The Netherlands; 2 Amsterdam Center for the Study of Adaptive Control in Brain and Behaviour (ACACIA), Department of Psychology, Universiteit van Amsterdam, Amsterdam, The Netherlands; University of Granada, Spain

## Abstract

Chronic use of cocaine is associated with impairment in response inhibition but it is an open question whether and to which degree findings from chronic users generalize to the upcoming type of recreational users. This study compared the ability to inhibit and execute behavioral responses in adult recreational users and in a cocaine-free-matched sample controlled for age, race, gender distribution, level of intelligence, and alcohol consumption. Response inhibition and response execution were measured by a stop-signal paradigm. Results show that users and non users are comparable in terms of response execution but users need significantly more time to inhibit responses to stop-signals than non users. Interestingly, the magnitude of the inhibitory deficit was positively correlated with the individuals lifetime cocaine exposure suggesting that the magnitude of the impairment is proportional to the degree of cocaine consumed.

## Introduction

Since a couple of years, because of the sinking prize in the European market [1], cocaine is not an “elite” drug anymore but is affordable for everyone, especially for purpose of recreational use. It is therefore likely that in the next years the recreational use of cocaine will become a public health issue, as is currently also the case for the recreational use of ecstasy [1].

At long term, chronic use of cocaine is associated with a reduced functioning of Dopamine D2 (DAD2) receptors [2] and dysfunctions in lateral prefrontal cortex (LPFC), in anterior cingulate cortex (ACC), as well as in orbitofrontal cortex (OFC) [3], [4]. Given that all these areas have been shown to play major roles in the control of goal-directed action [5], cocaine dependence is assumed to be correlated with deficiencies in cognitive control functions [6], [7]. Indeed, a majority of studies on chronic cocaine users (see [3], [8] for a review) points in that direction: Chronic users, compared to non-users, show a poorer ability to inhibit their overt responses [9], perform worse on tasks measuring mental flexibility [6], show compromised ability to control their attention [10], and choose disadvantageously in a decision-making task [11]. Particularly strong seems to be the link between long-term cocaine use and impairments of inhibitory control processes [1], [3], [12], [13]. This fits with the proposed crucial role of frontal lobe circuits in the inhibition of prepotent responses [14] and with the assumption that these circuits are innervated by dopamine [15]-the transmitter targeted by cocaine consume. However, the relation between inhibitory control functions and cocaine is complicated by possible pre-existent neuro-developmental factors. Recent evidence showed that subjects having preexisting lowered D2 receptor densities demonstrate higher risks to use cocaine and to become addicted [16] and that chronic users may suffer pre-existing problems in inhibitory control [17].

The aim of this study was twofold. First, we were interested to see whether recreational cocaine use is associated with impairments in inhibitory control to a significant degree. A “chronic” user, as described in the existing literature, consumes cocaine (preferably by smoking route, the so called “crack”) on a very regular base (1 gram daily, or at least 3 gram weekly) meets the Diagnostic and Statistical Manual of Mental Disorders (DSM-IV) [18] criteria for cocaine dependence or abuse. So far, however, no studies have systematically looked into inhibitory control impairments in the upcoming type of recreational user, who does not meet the criteria for abuse or dependence but takes cocaine (preferably by snorting route) on a monthly frequency (1 to 4 gram, which however is commonly consumed in only a few sessions, so that the peak use [bingeing] often equals this monthly dose). Bolla et al. [3] and Verdejo-Garcia et al. [19] considered that the magnitude of cognitive impairments may be proportional to the amount cocaine consume, which would suggest, first, a positive correlation between lifetime cocaine exposure and impairment in inhibitory control and, second, that recreational users do show impaired inhibitory control but to a smaller extent than reported for chronic users.

A second aim of this study was to improve on the experimental method. Previous studies on cocaine use suffer from numerous methodological shortcomings and confounds, such as inadequate screening procedures and controls for age, race, gender distribution, and level of intelligence, lack of a control group, and more, which makes it difficult to draw firm conclusions from the available data (see [3], [8] for a review). The design of the present study aimed at fixing these shortcomings.

Hence, the present study tested, by means of the well-established stop-signal task [20], whether the recreational intake of cocaine, strictly controlled for confounds, produces deficiencies of inhibitory control. In the standard stop signal task [21], participants are first presented with a stimulus that signals the execution of a particular (overt or cognitive) response, which may (or may not) be followed by a stop signal calling for the immediate abortion of that response. Versions of this task have been used to investigate the efficiency to stop various sorts of cognitive processes and so performance on it can be considered to diagnose the individual efficiency of actively inhibiting one's “thoughts and actions” [18], [19].

Recent neuroimaging as well as lesion studies have provided compelling evidence for the involvement of the right inferior frontal cortex (rIFC) in the act of inhibiting responses in the stop signal paradigm [22], [23]. Individuals that stopped faster to stop signals displayed more activity in the rIFC as well as in the right subthalamic nucleus (STN), a region in the basal ganglia, compared to slower inhibitors. These findings were interpreted to suggest a neuroanatomical substrate of stop-signal inhibition, involving a loop between rIFC and STN (see also [24], [25])

In our version of the task [25], participants responded to the direction of a green arrow by pressing a button with the left or right index finger. The stop signal was a sudden and unpredictable change of the arrow to red, signalling a deliberate effort to refrain from responding. The performance in the stop-signal paradigm can be conceptualized in terms of a race, in which the stopping process and the go process compete to finish first [20]. If the stop process finishes before the go process, the response is inhibited. By contrast, if the go process finishes before the stop process, the response is executed. The stop-signal task measures both the efficiency of response execution (by means of reaction times to go-signals) and the efficiency in inhibitory control (by means of the stop signal reaction time or SSRT, where longer SSRT reflect general slowing of inhibitory processes and indicate a lower level of inhibitory efficiency). Following our reasoning, we expected that recreational users compared to cocaine-free controls would show a selective deficit in the ability to inhibit (longer SSRT) but not in the execution of response (comparable RT to go-signals) [see also [9], [26] for the observations of these pattern of results in chronic users]. These observations led us to expect first, a positive correlation between lifetime cocaine exposure and impairment in inhibitory control (which would indicate that the magnitude of performance difficulties is proportional to the degree of cocaine consume [3], [19]) and, second, that recreational users did show impaired inhibitory control but to a smaller extent than reported for chronic users [9].

## Results

First, we tested group differences by means of t-tests. Analyses of mean RT to go-signals showed that recreational users of cocaine (382 ms) did not react significantly faster than cocaine-free controls (391 ms), *F*<1. This is consistent with our expectation that cocaine users and cocaine-free controls would exhibit comparable performance with respect to response execution.

**Figure 1:**
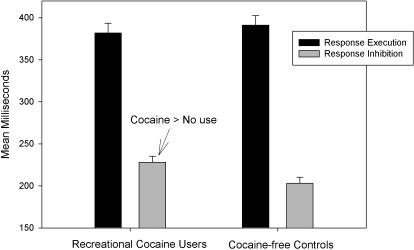
Mean go-signal RT (response latency) and mean SSRT (stopping latency) for recreational cocaine users and cocaine-free controls. Vertical capped lines atop bars indicate standard error of the mean.

Second, SSRTs were computed for each participant and for each group separately. The data of one male cocaine-free control was excluded because he failed to inhibit in more than 65% of the trials. The data of one male recreational users was excluded because after the saliva sample test he reported to be under the acute effect of cocaine. All other participants were able to stop their responses on stop-signal trials successfully in about half of the time a stop signal instructed them to do so (48% in users and 50% in non users), indicating that the dynamic tracking algorithm worked well in both groups. The percentage of choice errors to go-signals was low and did not discriminate between recreational users (1.9%) and cocaine-free users (1.0%). Most importantly, SSRT was significantly longer for users (228 ms) than for non users (203 ms), *t*(22) = 2.41, *p* = .025, see Figure 1. Interestingly, the size of this inhibitory deficit in recreational users (25 ms) was smaller than what has been reported for chronic users (65 ms) [9]. We further tested whether alcohol and cigarettes consumption contributed to the effect on SSRTs. However, an ANOVA with group as independent variable and monthly drinks and cigarettes as covariates did not point out such contribution: the effects of the covariates was far form significant, for both *F*<1 , and the group effect remained reliable, *F*(1, 23) = 4.25, *p* = .05.

**Figure 2:**
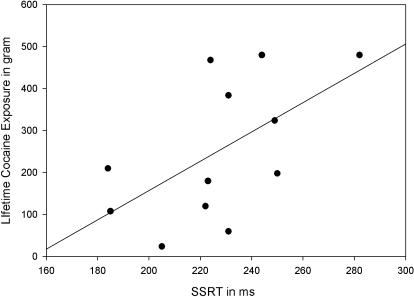
Scatter diagram of individual lifetime cocaine exposure (in gram) against SSRT (in ms).

Third, to test whether the magnitude of cognitive impairments is proportional to the amount of cocaine consumed and/or to alcohol and tobacco use, we computed Pearson correlation coefficients between the individual lifetime cocaine exposure, peak and monthly cocaine dose, monthly drinks and cigarettes and SSRT. Lifetime cocaine exposure positively correlated with SSRT, *r*(12) = .625, *p* = .05, while peak and monthly cocaine dose and monthly drinks and cigarettes, even though it followed the same trend, did not. Hence, longer cocaine exposure is associated with less efficient inhibitory control, see Figure 2.

## Discussion

This study tested, for the first time, whether the recreational use of cocaine is associated with a detectable selective impairment in the ability to inhibit responses. Our findings suggests an affirmative answer: recreational users showed normal response speed but impaired inhibitory control, and the size of this deficit seems to correspond to the amount of cocaine consume [3], [19]. Hence, the greater the dose and the frequency of cocaine use, the greater the magnitude of the loss of inhibitory control seems to be. In view of evidence suggesting that cocaine is accompanied by a selective effect on DAD22, our findings are consistent with the hypothesis that dopamine modulates response inhibition [15].

In contrast to numerous previous studies of chronic cocaine users, the design of our study allows us to reject a number of alternative accounts of our observations. Participants were screened for several psychiatric disorders and matched for age, IQ, sex, and alcohol consumption, which rules out accounts in terms of pre-existing psychiatric disorders (as schizophrenia, ADHD, and obsessive compulsive disorder) that are known to affect response inhibition [27], [28], [29]. Particularly important was the matching of the age range: While inhibitory control seems not to be related to general intelligence [21], there is evidence that cognitive inhibitory process declines throughout the life span [21]. Given that MDMA is associated with impairments in working memory processes and cannabis is related to dysfunctions in cognitive flexibility and that both drugs seem not to be linked with malfunction in inhibitory control function [19], we doubt that our results can be attributed to the use of marijuana and MDMA.

Given the seemingly small amount of cocaine involved, the present findings are worrying. Even though the task we used to diagnose the inhibitory deficiency in recreational users is rather artificial, the deficit itself is likely to affect everyday behavior. Many real-life situations require the active inhibition of a pre-potent action. This is particular obvious for examples like traffic behavior, where stopping to walk or to drive is necessary when the traffic light turns from green to red, or when passengers, animals, or vehicles are suddenly crossing the street.

The present findings raise the question whether recreational cocaine users also show impairments in other cognitive control functions, such as the shifting between tasks and mental sets, and the updating and monitoring of working memory [30]. The direct effects of recreational cocaine use on the brain need to be explored as well. It remains to be demonstrated, for instance, that recreational use of cocaine produces changes at the neuromodulatory (reduced functioning of DAD2 receptors) and functional level (dysfunction in LPFC, ACC, and OFC) that are proportional to the degree of behavioural performance deficits.

Moreover, the findings obtained in this study are important because they spot a selective behavioral deficit that could contribute to cocaine use, and explain its association with other disorders of impulse control, such as ADHD and pathological gambling. However, their etiological role in cocaine use are still uncertain, and more research is necessary to determine the relative contribution of cocaine use and other pre-existing constellation in the creation of inhibitory control impairment.

## Materials and Methods

### Participants

Twenty-six young healthy adults served as participants for partial fulfilment of course credit or a financial reward and constituted the two groups: recreational users of cocaine and cocaine-free controls. Participants were recruited via notes posted on community bulletin boards and by word of mouth. Recreational users of cocaine met the following criteria: 1) a monthly consumption (1 to 4 gram) by snorting route for a minimum of two years; 2) no Axis 1 psychiatric disorder (DSM-IV) [18], including ‘substance abuse’; 3) no clinically significant medical disease; 4) no use of medication. Cocaine free-controls met the same criteria expect that they reported no history of past or current cocaine use. Subjects were selected by means of a phone interview by a research assistant with the M.I.N.I. [31], a brief diagnostic tool that screens for several psychiatric disorders including, among others, schizophrenia, depression, mania, ADHD, and obsessive-compulsive disorder. Participants with a known history of psychopathology and those who were taking medication were excluded. Participants were asked to refrain from taking drugs for two days and from all caffeine containing foods and beverages for 12 hours prior to the experimental sessions, not to consume alcohol on the night before the experimental session and to have a normal night rest. Subjects' compliance with the instruction was encouraged by taking a saliva sample (not further analyzed) at the beginning of the session [32], [33], [34].

In the last month six of the thirteen recreational users and two of the thirteen cocaine-free users also smoked marijuana, while four recreational users reported to have taken one MDMA (ecstasy) tablet. Participants in the two groups were matched for race (100% Caucasian), age, sex and IQ (measured by Raven's Standard Progressive Matrices [SPM] [35]) and alcohol consumption. Demographic and drug use statistics are provided in Tables 1 and 2.

**Table 1:** Demographic characteristics

Sample	Controls	Recreational users	Significant
*N (M∶F)*	13 (11∶2)	13 (11∶2)	ns
*Age (years)*	29.0 (9.1)	29.0 (6.5)	ns
*Raven IQ*	115.3 (3.6)	111.1 (5.0)	ns
*Monthly drinks*	53.4 (61.5)	87.3 (77.2)	ns
*Monthly cigarettes*	98.4 (260.4)	391.5 (193.3)	*

Ns, Non-significant difference; Raven IQ, IQ measured by means of the Raven Progressive Matrices; monthly drinks, monthly number of standard alcoholic drinks; monthly cigarettes, monthly number of cigarettes smoked per day.

* *p*<.01

**Table 2:** Self-reported use of cocaine

Sample	Mean (SD)
*Highest regular frequency (times per month)*	3.1 (2.6)
*Highest amount in a 12-h period (peak; grams)*	1.2 (0.7)
*Monthly grams*	2.3 (1.1)
*Lifetime exposure grams*	253 (168)
*Mean abstinence duration (days)*	7 (8)
*Monthly money cocaine (Euro)*	117.3 (54.3)

Written informed consent was obtained from all participants after the nature of the study was explained to them; the protocol was approved by the institutional review board (Leiden University, Institute for Psychological Research), which approved the remuneration arrangements of 20 Euro.

### Apparatus and stimuli

Responses were made by pressing the “Z” or “?” of the QWERTY computer keyboard with the left and right index finger, respectively. Participants were required to react quickly and accurately by pressing the left and right key in response to the direction of a left- or right-pointing green arrow (go trials) of about 3.5×2.0 cm.

### Procedure and design

All participants were tested individually. During all sessions, participants provided a saliva sample, then, they completed the intelligence test and the stop-signal task.

Individual IQs were determined by means of a 30-min reasoning-based intelligence test (Raven's Standard Progressive Matrices: SPM [35]). Each item of this test consists of a pattern or sequence of a diagrammatic puzzle with one piece missing, the task being to complete the pattern or sequence by choosing the correct missing piece from a list of options. The items are getting more difficult as the test taker proceeds through the test. The SPM assesses the individual's ability to create perceptual relations and to reason by analogy independent of language and formal schooling; it is a standard, widely-used test to measure Spearman's g factor and of fluid intelligence in particular [35].

The stop-signal task consisted of a 30-min session [25]. Arrows were presented pseudorandomly, with the constraint that they signaled left- and right-hand responses equally often. Arrow presentation was response-terminated. Participants were required to react quickly and accurately by pressing the left and right key in response to the direction of a left- or right-pointing green arrow (go trials) of about 3.5×2.0 cm. Intervals between subsequent choice signals varied randomly but equiprobably, from 1250 to 1750 ms in steps of 125 ms. During these interstimulus intervals, a white fixation point (3 mm in diameter) was presented. The green arrow changed to red on 30% of the trials, upon which the go response had to be aborted (stop trials). A staircase-tracking procedure dynamically adjusted the delay between the onset of the go signal and the onset of the stop signal to control inhibition probability [36]. After a successfully inhibited stop trial, stop-signal delay on the next stop trial increased by 50 ms, whereas the stop-signal delay decreased by 50 ms on the next stop trial when the participant was unable to stop. This algorithm ensured that motor actions were successfully inhibited in about half of the stop trials, which yields accurate estimates of stop-signal RT [37] and compensates for differences in go-signal RT between participants and groups. The stop task consisted of five blocks of 104 trials each, the first of which served as a practice block to obtain stable performance.
